# pH-responsive graphene oxide loaded with targeted peptide and anticancer drug for OSCC therapy

**DOI:** 10.3389/fonc.2022.930920

**Published:** 2022-08-03

**Authors:** Ran Li, Ruifang Gao, Yingjiao Zhao, Fang Zhang, Xiangyu Wang, Bing Li, Lu Wang, Lixin Ma, Jie Du

**Affiliations:** ^1^ Department of Preventive Dentistry, Shanxi Medical University School and Hospital of Stomatology, Taiyuan, China; ^2^ Shanxi Province Key Laboratory of Oral Diseases Prevention and New Materials, Shanxi Medical University School and Hospital of Stomatology, Taiyuan, China; ^3^ Research Division/Biomolecular Imaging Center, Harry S. Truman Memorial Veterans’ Hospital, Columbia, MO, United States; ^4^ Department of Radiology, University of Missouri, Columbia, MO, United States

**Keywords:** oral squamous cell carcinoma, graphene oxide, pH-responsive drug release, anti-cancer drug, gastrin releasing peptide receptor

## Abstract

Oral squamous cell carcinoma (OSCC) is the most common type of cancer occurring in the oral and maxillofacial regions. Despite of the advances in the diagnosis and treatment, the overall 5-year survival rate has remained about 40%–50% in the past decades. Various nanotechnology-based carrier systems have been investigated for their potentials in the OSCC treatment. However, because of the lack of active targeting of tumors, their application is limited. Studies have shown that gastrin-releasing peptide receptors (GRPRs) are overexpressed on many human cancers, including head and neck squamous cell carcinoma. Herein, we aimed to develop a GRPR-targeted nano-graphene oxide (NGO) nanoprobe drug delivery system for OSCC therapy. DOX@NGO-BBN-AF750 was synthesized by the non-covalent bonding method to couple carboxylated NGO with BBN-AF750 (bombesin antagonist peptides conjugated to Alexa Fluor 750) and DOX (doxorubicin) through π-π and hydrogen bonding. Internalization and antitumor activities were carried out in human HSC-3 cancer cells. The tumor pH microenvironment was simulated to study the release of antitumor drug DOX from the DOX@NGO-ant BBN-AF750 complex under different pH conditions. DOX@NGO-BBN-AF750 showed internalization into HSC-3 cells. The IC50 (50% inhibitory concentration) was 5 µg/ml for DOX@NGO-BBN-AF750 in HSC-3 cells. Furthermore, DOX@NGO-BBN-AF750 showed a pH-sensitive drug release rate, and a dose-dependent and pH-responsive cytotoxicity in HSC-3 cells. DOX@NGO-BBN-AF750 presents the characteristics ensuring a slow release of DOX from the nanoprobe, thereby protecting the drug from degradation and prolonging the half-life of the drug. This report provides a versatile strategy to achieving targeted and imaging-guided therapy of OSCC.

## Introduction

Oral cancer is one of the most prevalent cancers worldwide, presenting a global incidence of more than 377,713 new cases and 177,757 deaths every year (1). Oral squamous cell carcinoma (OSCC), the most common oral cancer subtype, constitutes more than 90% of all oral cancers (2). Despite of the recent advances in clinical treatment of OSCC, the prognosis of patients with OSCC remains poor with a 5-year survival rate of 40%–50% (3, 4). Thus far, chemotherapy is still the mainstream treatment for advanced OSCC whenever salvage surgery or re‐irradiation is not feasible. To improve the therapeutic efficacy for OSCC, combinational treatment of surgery with radiotherapy or chemotherapy is a commonly adopted strategy (5). Although conventionally combinational chemotherapy can significantly improve the therapeutic efficacy, it also brings severe adverse side effects to patients and increases burden on healthcare systems (6). Most anticancer drugs are non-selective, which leads to damage of healthy cells or tissues apart from targeting cancer cells and further results in multidrug resistance during treatment due to additions of drugs that lack specificity (7). In recent years, targeted therapies have shown promising results to combat cancer progression (8). During the past few decades, nanocarriers as drug delivery systems have attracted more and more attention in the research field (9–11). Nanodrugs have been shown to reduce the side effects of cancer chemotherapy and improve the treatment efficacy (12–14).

In the previous few years, the growing exploration of nanomedicine has contributed greatly to cancer treatment (15). Graphene consists of two-dimensional sheets of sp2-hybridized carbon with a honeycomb-like structure, while one out-of-plane p orbital that provides the electron delocalization network (16, 17). This inherent property of the graphene structure makes it a fictile drug carrier and transporting system. However, being a water-insoluble material limits the biomedical applications of graphene. On the other hand, graphene oxide (GO) of the graphene derivatives possesses appropriate expanded surface areas and abundant functional groups, such as hydroxyl (-OH), ring oxides (-O-), and carboxyl groups (-COOH), for easiness of surface functionalization and biocompatibility, which has attracted extensive attention in the drug delivery field (18, 19). In addition, as a kind of near infrared (NIR) light-absorbing nanomaterials, GO shows a high photothermal conversion efficiency in the NIR region (20, 21). For example, Zhang et al. reported a dual-sensitive GO loaded with proapoptotic peptides and anticancer drugs for cancer synergetic therapy (22). In solid tumors, the existence of capillary endothelial cell fenestrations results in high permeability of macromolecular drugs, and the absence of lymphatic capillaries leads to the retention of macromolecular drugs, *via* the enhanced permeability and retention (EPR) effect (23–25). Generally, the nanocarriers used as drug delivery systems are mainly designed based on a passive mechanism (26). However, because of the lack of active targeting to tumors, which is necessary for improvement of tumor diagnosis and treatment, their application has been limited (27). The surface modification to include targeted ligands, which can be antibodies, peptides, or aptamers, is a viable strategy to improve the nanodrugs’ ability of intracellular delivery of the drug payload to tumor cells (18, 28). Tian et al. showed that GO conjugated with pegylated folate (FA-PEG-GO) and loaded with anticancer drugs was selectively taken up into the lysosomes of cancer cells through folate receptor-mediated endocytosis, and the acidic environment caused the drug release to induce apoptosis (29). Howard et al. generated actively targeted nanomaterials by conjugating PEGylated NGO to a bispecific antibody (BsAb) with dual specificity and demonstrated that compared with non-targeted nanomaterials, the antibody-targeted nanostructures had improved accumulation in tumor cells (30). In another study, hyaluronic acid (HA), a ligand to the hyaluronan receptor CD44, was conjugated onto GO for a targeted system loaded with metformin (HA-GO-Met); the *ex vivo* and *in vivo* tumor regression study showed that HA-GO-Met could induce apoptosis for triple-negative breast cancer (31).

Gastrin-releasing peptide receptor (GRPR) has been shown to be overexpressed on many tumors, such as human breast cancer, prostate cancer, colon cancer, and cervical cancer (32–37). Lango et al. demonstrated that GRPR is overexpressed in squamous cell carcinoma of the head and neck (HNSCC), and GRPR was further used as a biomarker for surgical margin prediction in a murine orthotopic model of oral cancer (38). Bombesin (BBN), an amphibian homolog of mammalian gastrin-releasing peptide (GRP), is a 14-amino acid polypeptide with a C-terminal eight-amino acid sequence Gln-Trp-Ala-Val-Gly-His-Leu-Met that specifically binds to GRPR (39–41). BBN (1–14) and its derivatives have been extensively used for the development of molecular probes for imaging of GRPR on tumors (42, 43). However, natural BBN (1–14), a receptor agonist, binds to GRPR to promote the proliferation of tumor cells and causes side effects such as gastrointestinal discomfort (44, 45). In recent years, receptor antagonists have been developed and studied by modifications on the C terminal amino acids of BBN for the diagnosis and treatment of tumors (46, 47). We have previously shown that BBN antagonist peptides conjugated to Alexa Fluor 750 (BBN-AF750) have high binding affinity and specificity to GRPR-overexpressed tumors (46), including human OSCC biopsies and HSC-3 cells (48). We further demonstrated that nano-graphene oxide (NGO) coupled with BBN-AF750 peptides (NGO-BBN-AF750) specifically targets GRPR in human HSC-3 cells (48).

In this study, we aim to investigate whether the GRPR-targeted NGO nanoprobe is a potential OSCC-targeted drug carrier for loading and pH-sensitive release of anticancer drugs. We constructed a GRPR-targeted nanodrug delivery system, DOX@NGO-BBN-AF750, by the non-covalent bonding method to couple carboxylated NGO with BBN-AF750 and doxorubicin (DOX) through π–π and hydrogen bonding. We investigated the cell internalization and antitumor activities of the targeted drug delivery system *in vitro*. The tumor pH microenvironment was simulated to study the release of antitumor drug DOX from the DOX@NGO-BBN-AF750 complex under different pH conditions. CCK8 assay was used to determine the effect of DOX on the proliferation of HSC-3 cells.

## Materials and methods

### Materials

Carboxylated graphene oxide (GO-COOH) sheets were purchased from Nanjing Xianfeng Nano Material Tech Co., Ltd. Doxorubicin hydrochloride (DOX·HCl) was obtained from Boster Biological Technology, China. AF750-6Ahx-Sta-BBN was synthesized and purified at the University of Missouri, Columbia, MO, USA, according to the published procedure (46) (38).

### Cell lines and culture conditions

The HSC-3 cells (human tongue squamous cell carcinoma cell line), obtained from the American Type Culture Collection (ATCC, Rockville, MD), were cultured in Dulbecco’s Modified Eagle’s Medium (DMEM, GIBCO) consisting of 10% fetal bovine serum (FBS; GIBCO) and 1% penicillin–streptomycin (GIBCO). The cells were cultured in a 5% CO_2_ atmosphere at 37°C.

### Preparation of BBN antagonist-based tumor-targeting and pH-sensitive nanoparticles

First, NGO-BBN-AF750 nanoprobes were synthesized and purified according to the procedures in our previous article (48). Briefly, NGO-COOH and AF750-6Ahx-Sta-BBN were added in Tris–HCl buffer, stirred for 40 min in the dark, and centrifuged, and the supernatant was removed to obtain NGO-BBN-AF750.

To synthesize DOX@NGO-BBN-AF750, we weighed 10 mg of carboxylated NGO-BBN-AF750, dissolved in 10 ml of ultrapure water, and sonicated; 0.5 ml of 1 mg/ml DOX was added, reacted for 2 h, and centrifuged at 10,000 rpm × 10 min; and the supernatant was removed and finally freeze-dried to obtain final nanoparticles. The drug loading efficiency (DLE) of DOX@NGO-BBN-AF750 was determined *via* the following equation:


$$DLE\;\left(wt\;\%\right)=\;\frac{m_{total}-m_{unload}}{M}\times \;100\%$$

where M is the weight of the added DOX, and *m_total_* and *m_unload_* are the nanoparticle weights after and before DOX loading, respectively.

### Characterizations

The nanoparticles were analyzed using a Fourier transform infrared (FT-IR) spectrometer (TENSOR 27, Germany). Specifically, the nanoparticles and KBr were evenly mixed in the ratio of 1:100 to form powder and pressed into tablets, and the spectrum was collected with a resolution of 0.25 cm^−1^ between the wave numbers of 400 and 4,000 cm^−1^. Furthermore, ultraviolet–visible (UV–Vis) spectroscopic measurements were performed using UV-3600 (Shimadzu, Japan) and absorption spectra were recorded within the range of 200–1,200 nm at room temperature. Fluorescence spectroscopy was recorded with fluorescence intensity at λex = 740 nm and λem = 900 nm with Ex. slit (nm) = 10. Transmission electron microscopy (TEM) was performed on JEOL, and zeta potential was determined using JEM-2100F (JEOL Ltd., Japan). Moreover, the morphology of DOX@NGO-BBN-AF750 was examined using a field emission scanning electron microscope (FE-SEM; S4800; Hitachi, Tokyo, Japan) at an accelerating voltage of 1 kV through gold sputter coating.

The intensities of the UV–Vis absorption peaks were estimated from the integrated area of each peak using Origin (https://www.originlab.com). The relative ratio of each peak was obtained to estimate the amount of BBN-AF750 on DOX@NGO-BBN-AF750. Briefly, the UV–Vis spectra of 0.1 mg/ml NGO, 50 nM BBN-AF750, 0.1 mg/ml NGO-BBN-AF750, and 0.1 mg/ml DOX@NGO-BBN-AF750 were acquired. The intensity of the NGO absorption peak at 240 nm was measured and normalized to 0.1 mg/ml NGO. Next, the AF750 peak at 750 nm was measured. The amount of BBN-AF750 was determined by comparing with the 50-nM BBN-AF750 absorption peak.

To determine the *in vitro* stability of the nanoparticles in physiological conditions, we dissolved NGO, NGO-BBN-AF750, and DOX@NGO-BBN-AF750 in deionized water and 10% FBS (DOX: 5 µg/ml) and then observed their dispersity and stability at 0, 12, 24, 48, and 72 h, respectively.

### *In vitro* DOX release from DOX@NGO-BBN-AF750

The DOX *in vitro* release experiments were performed in PBS buffers at different pH (5.6, 6.6, 7.4) at 37°C. Briefly, 2 mg DOX@NGO-BBN-AF750 was dispersed in 2 ml ultrapure water, dialyzed (MECO 8,000–14,000 Da) in a dark environment, and then infiltrated into 98-ml PBS buffers of different pH and tested under continuous shaking at 37°C. At certain time points, 2 ml of each of the above buffers was taken out and replaced with equivalent fresh PBS buffer. The amount of drug released was measured by UV–Vis spectroscopy (DOX: 480 nm) and calculated from a standard curve. The cumulative drug release is calculated as follows (49):


$$Cumulative\;release\;\left(\%\right)=\;\frac{2\times \sum_{i=1}^{n-1}Ci+100\times Cn}{weight\;of\;drug\;in\;DOX@NGO\_BBN\_AF750}\times \;100\%$$

where *Ci* is the concentration of DOX drug in dialysate at time *i* and *Cn* is the concentration of the DOX drug in PBS at the last time point.

### *In vitro* antitumor activity of DOX@NGO-BBN-AF750

First, we tested the effect of DOX on HSC-3 cell proliferation through CCK-8 assay (BOSTER, Wuhan, China). Briefly, HSC-3 cells were seeded in 96-well plates at a density of 5,000 per well and incubated for 24 h. DOX at different concentrations (1, 2, 4, 8, 16 µg/ml) was added and incubated for 24 h, followed by further incubation with 10 µl of CCK-8 for 30 min. Finally, the absorbance was measured at 450-nm wavelength using UV–Vis spectroscopy (UV-3600, Shimadzu, Japan).

For cell uptake assay, we conducted confocal laser scanning microscopy (CLSM) observations. HSC-3 cells were cultured in 24-well plates at 2 × 10^4^ cells/well and grown for 24 h. Next, the original medium was replaced with fresh medium containing either 50 nM AF750-6Ahx-Sta-BBN and 5 µg/ml DOX or 105 µg/ml DOX@NGO-BBN-AF750. The cells were then incubated for 4 h. After washing the cells three times with PBS, 2.5% paraformaldehyde was used to fix the cells. Then DAPI (excitation wavelength: 488 nm) was used to stain the nuclei of the cells. Finally, the cells were visualized under OLYMPUS FV1200 confocal laser scanning microscope (Olympus, Osaka, Japan) in the DAPI (for nuclei, Ex. 370 nm, Em. 480 nm) and the near-infrared fluorescence (for BBN-AF750, Ex. 710 nm, Em. 790 nm) wavelength windows.

The cell viability test was performed using free DOX, NGO, BBN-AF750, NGO-BBN-AF750, or DOX@NGO-BBN-AF750 against HSC-3 cells by CCK-8 assay. Briefly, HSC-3 cells were seeded in 96-well plates at a density of 5,000 per well for 24 h. The original medium was replaced with fresh medium, and DOX, NGO, BBN-AF750, NGO-BBN-AF750, and DOX@NGO-BBN-AF750 (DOX: 5 µg/ml) were added, respectively. After being incubated for another 24 h, the medium was replaced with 90 µl fresh medium containing 10 µl CCK-8 for 30 min. Moreover, the absorbance was measured at 450 nm. In addition, we examined the effects of free DOX or DOX@NGO-BBN-AF750 on the proliferation of HSC-3 cells under different pH (5.6, 6.6, and 7.4) conditions.

### Statistical analysis

Data are presented as mean ± standard deviation. Comparisons between experimental and control groups were performed using Student’s *t*-test. Significance is defined with *P* values less than 0.05.

## Results

### Characterization of NGO-BBN-AF750 and DOX@NGO-BBN-AF750

**Figure 1:**
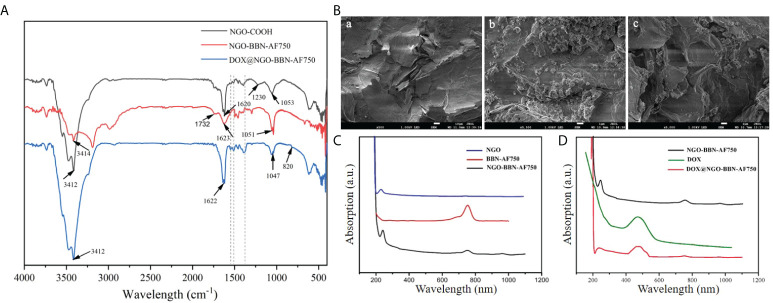
Characterizations of the nanoprobes. **(A)** Fourier transform infrared (FT-IR) spectroscopy of NGO-COOH, NGO-BBN-AF750, and DOX@NGO-BBN-AF750. **(B)** SEM images of NGO (a), BBN-AF750 (b), and DOX@ NGO-BBN-AF750 (c). Scale bars: 10 µm. **(C)** UV–Vis spectra of NGO, BBN-AF750, and NGO-BBN-AF750. **(D)** UV–Vis spectra of DOX, NGO-BBN-AF750, and DOX@NGO-BBN-AF750.

We first synthesized NGO-BBN-AF750 and DOX@NGO-BBN-AF750 by the non-covalent bonding method and purified with centrifugation. The nanoprobes were characterized by FT-IR spectroscopy, TEM and SEM microscopy, and UV–Vis spectrophotometry (**Figure 1**). As shown in **Figure 1A**, the characteristic stretching OH bands were observed around 3,412–3,414 cm^-1^ for NGO-COOH, NGO-BBN-AF750, and DOX@NGO-BBN-AF750. The vibration peaks at 1,620–1,623 cm^-1^ due to the C=C stretch were found in all three nanoparticles. Particularly, the C–O–C absorption peak was found at 1,230 cm^-1^ in NGO-COOH, but not in NGO-BBN-AF750 and DOX@NGO-BBN-AF750. The disappearance of the 1,230-cm^-1^ C–O–C peak indicates alterations in the C–O–C bond by the formation of a hydrogen bond and π–π bond interactions upon coupling with BBN-AF750 and DOX. Furthermore, the C=O carboxyl vibration peak at 1,732 cm^-1^ was not observed in DOX@NGO-BBN-AF750. The reason may be the formation of hydrogen bond due to the coupling of DOX weakening the C=O stretching vibration. Moreover, new peaks of the aromatic ring C=C stretches for DOX were observed at 1,560 and 1,515 cm^-1^ in DOX@NGO-BBN-AF750 (50). All the above FT-IR results establish the formation of DOX@NGO-BBN-AF750.

Moreover, the microstructure of NGO-BBN-AF750 was observed under a transmission electron microscope. Furthermore, we observed the surface morphology of DOX@NGO-BBN-AF750 by SEM. In comparison with NGO [**Figure 1B(a)**], SEM showed a small granular structure on the surface of NGO-BBN-AF750 [**Figure 1B(b)**], which may be BBN-AF750. We further found wrinkles and curls on DOX@NGO-BBN-AF750 [**Figure 1B(c)**]. In addition, a zeta potential change from -21.4 to -16.6 and -14.4 Mv was observed in NGO, NGO-BBN-AF750, and DOX@NGO-BBN-AF750, respectively, as shown in **Table 1**.

**Table 1:** Zeta potentials.

Sample	Zeta potential (Mv)
NGO	-21.44 ± 039
NGO-BBN-AF750	-16.56 ± 0.8
DOX@NGO-BBN-AF750	-14.64 ± 0.8

DOX loading on NGO-BBN-AF750 was determined through measuring the absorbance using UV–Vis spectrophotometry. The UV–Vis spectra of NGO, BBN-AF750, and NGO-BBN-AF750 (**Figure 1C**) showed that NGO-BBN-AF750 had the appearance of the peak at the NIR wavelength 760 nm relative to that of NGO, indicating the surface modification of the NGO with the BBN-AF750 peptides. The UV–Vis spectrum of DOX@NGO-BBN-AF750NGO presented an additional peak at 480 nm (DOX) relative to that of NGO-BBN-AF750 (**Figure 1D**), further indicating the successful loading of DOX in DOX@NGO-BBN-AF750. The drug loading efficiency, DLE (wt%), of DOX in DOX@NGO-BBN-AF750 was calculated to be about 90%. The nanoparticle weight gain is 4.5% after DOX loading onto DOX@NGO-BBN-AF750.

DOX@NGO-BBN-AF750 exhibited good water dispersity. The size and overall nanoparticle morphology of DOX@NGO-BBN-AF750 were similar to those of NGO and NGO-BBN-AF750 observed under TEM (48). The nanoparticles appeared as two-dimensional flacks with the diameter range of 0.5–5 µm and thickness of 0.8–1.2 nm.

To determine the amount of BBN-AF750 on DOX@NGO-BBN-AF750, the intensity of the NGO absorption peak at 240 nm on UV–Vis was measured and normalized to 0.1 mg/ml NGO. Next, the AF750 peak at 750 nm was measured. The amount of BBN-AF750 was determined to be 7.6 nM in 0.1 mg/ml DOX@NGO-BBN-AF750.

### The pH-responsive release *in vitro*

**Figure 2:**
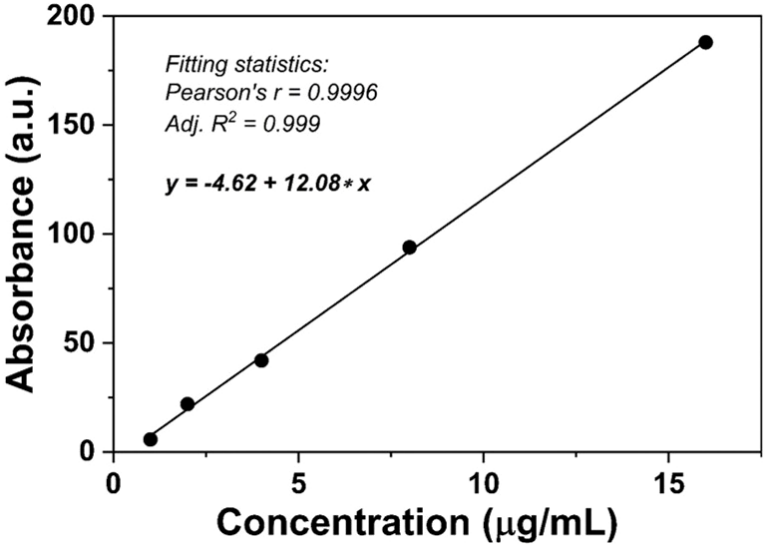
Standard curve of DOX absorbance as a function of concentration.

In DOX@NGO-BBN-AF750, DOX was attached to the surface or edge of GO *via* hydrogen bond and π–π bonds. Due to the differences in physiological environment between tumors and normal tissues, we set up different pH values to mimic the normal physiological tissue and tumor microenvironment condition. To quantitatively investigate the release of DOX from DOX@NGO-BBN-AF750, we first conducted the standard curve of DOX absorbance as a function of concentration as shown in **Figure 2**.

**Figure 3:**
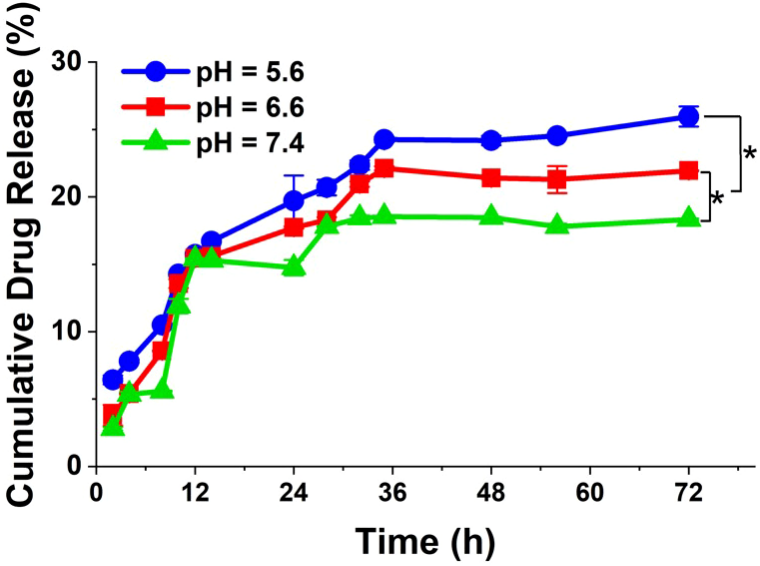
*In vitro* release profiles of DOX from DOX@NGO-BBN-AF750 under different pH values at 37°C. Data were obtained from n = 3 repeated experiments. *P < 0.05.

*In vitro* DOX release profiles from DOX@NGO-BBN-AF750 are shown in **Figure 3**. As demonstrated during the entire drug release process (up to 72 h), at pH 5.6 the drug release rate was faster than the other two (pH 6.6 and 7.4). Moreover, within the initial 9 h, DOX was released very fast. After 36 h, the drug release rates tend to be flat. We observed that the cumulative released drugs in PBS solution was 19.8%, 17.7%, and 15.3% at 24 h, at pH 5.6, 6.6, and 7.4, respectively. These results demonstrated that DOX@NGO-BBN-AF750 is pH-sensitive and that DOX release from DOX@NGO-BBN-AF750 can be promoted in the acidic microenvironment in tumors, which could potentially reduce the drug side effects in normal tissues.

### Cellular uptake and internalization

**Figure 4:**
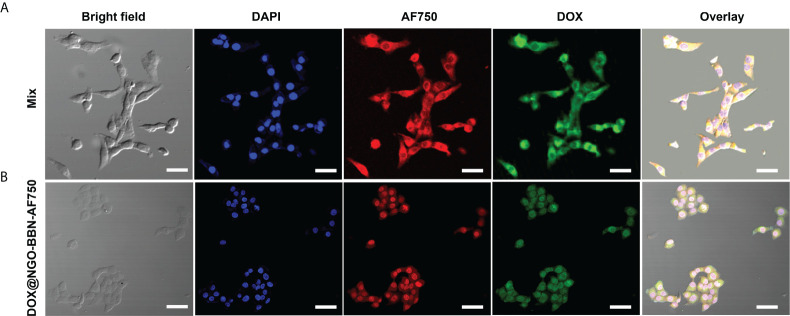
Microscopic images show cell uptake of **(A)** BBN-6Ahx-Sta-AF750 mixed with free DOX (mix group), and **(B)** DOX@NGO-BBN-AF750 in HSC-3 cells. The scale bar is 30 μm.

Because cell binding and internalization are a prerequisite for efficient administration of nanodrugs, the cellular uptake of DOX@NGO-BBN-AF750 was studied in HSC-3 cells using confocal laser scanning microscopy at three fluorescence channels, blue fluorescence (excitation, 460 nm) for nuclei, green color (excitation, 488 nm) for DOX, and NIR fluorescence (excitation, 750 nm) for AF750. As a control, HSC-3 cells treated with AF750-6Ahx-Sta-BBN mixed with free DOX were also studied. As shown in **Figure 4**, a strong cellular uptake was observed for both DOX and AF750 signals. However, AF750 appeared on the cell membrane of HSC-3 cells treated with free DOX and AF750-6Ahx-Sta-BBN (**Figure 4A**). In contrast, **Figure 4B** shows that DOX@NGO-BBN-AF750 was internalized into cells with strong AF750 and DOX fluorescence signals overlapping with nuclei compartment. Lammel et al. reported that GO penetrated cells by piercing and mechanically disrupting the plasma membrane and aggregated inside the cells (51). Studies have shown that the cellular uptake process of GO in cancer cells was *via* the mechanism of endocytosis in an energy-dependent process (52, 53). Our study shows that DOX@NGO-BBN-AF750 appeared in the cell nucleus after incubation of 4 h. Cellular internalization of DOX@NGO-BBN-AF750 could improve the intracellular delivery and utilization of anticancer nanodrugs.

### *In vitro* antitumor activity of the targeted drug delivery system

**Figure 5:**
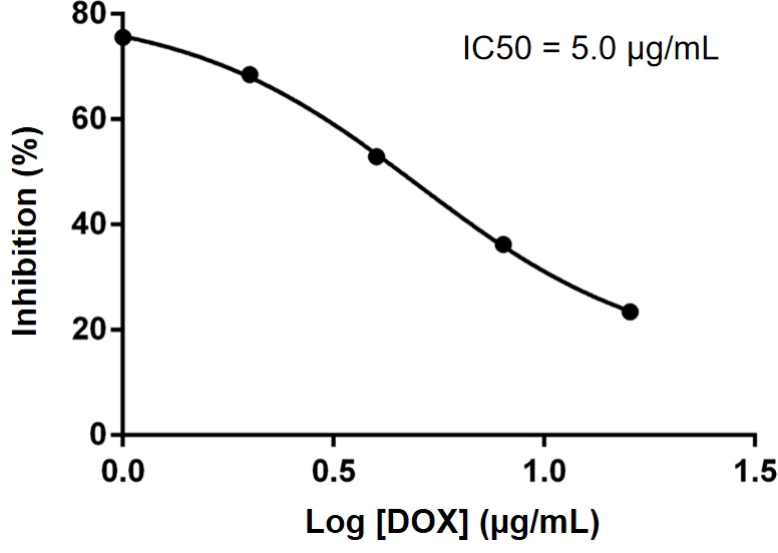
IC50 of DOX was determined to be 5.0 µg/ml in HSC-3 cells. Data were obtained from n = 5 measurements.

We investigated the effect of different concentrations of free DOX on the viability of HSC-3 cells by CCK8 assay and calculated its IC50. As shown in **Figure 5**, with the increase in DOX concentration, the activity of HSC-3 cells decreased, and the IC50 value was 5 µg/ml (Prism 6).

**Figure 6:**
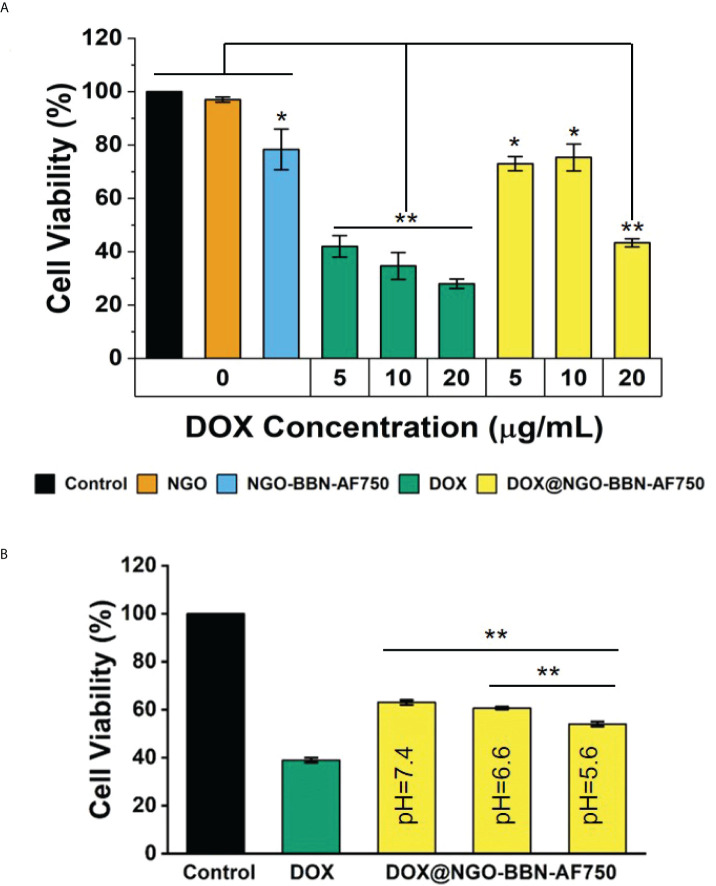
*In vitro* cytotoxicity of DOX and DOX@NGO-BBN-AF750 nanoprobes on HSC-3 cells. **(A)** Cell viability after treatment with NGO and NGO-BBN-AF750 or DOX and DOX@NGO-BBN-AF750 at different DOX concentrations (pH = 7.4). ** *P* < 0.01 compared with control, NGO, and NGO-BBN-AAF750 groups. *P < 0.05 compared with the control group. **(B)** Cell viability after treatment with DOX@NGO-BBN-AF750 at different pH environments (DOX = 5 µg/ml). Data were obtained from n = 5 measurements and are presented as mean ± SD. **P < 0.01.

CCK8 assays were performed in HSC-3 cells to evaluate the effects of our delivery systems on cell viability. The cytotoxicity of DOX and DOX@NGO-BBN-AF750 was tested on HSC-3 cells at the DOX concentrations of 5, 10, and 20 µg/ml. As a control experiment, cells were also treated with NGO or NGO-BBN-AF750 at the equivalent DOX@NGO-BBN-AF750 concentration. As shown in **Figure 6A**, no cytotoxicity was found with the NGO group, while a decrease in the cell viability with the NGO-BBN-AF750 group was likely due to the antagonist BBN targeting onto the cells (54–56). Notably, both free DOX and DOX@NGO-BBN-AF750 groups showed significant decreases in cell viability and a dose-dependent cytotoxicity response on HSC-3 cells. However, DOX@NGO-BBN-AF750 showed lower efficacy than free DOX, which can be explained by the incomplete release of DOX from the nanoparticle under the environment of pH 7.4. Indeed, further experiment demonstrated that as the pH of the culture medium decreased from pH 7.4 to 5.6, the cell viability of the DOX@NGO-BBN-AF750 groups decreased (**Figure 6B**). We further analyzed the pH effect in the control cells and the cells treated with DOX at the acidic pH of the cell medium. The control cells treated in pH 5.6 medium for 24 h showed cell viability around 88% which was lower than 100% at pH 7.4. However, the cells treated with 5 µg/ml DOX in pH 5.6 medium for 24 h showed no significant changes in cell viability compared to pH 7.4, where the cell viability was around 48% for pH 5.6 and 42% for pH 7.4. It has been reported that the acidic pH of the cell medium led to a decrease in DOX toxicity on cervical (Hela) and kidney (A498) cancer cell lines due to the change in drug permeability across the cell membrane as a result of drug protonation (57). In our study, DOX was carried by DOX@NGO-BBN-AF750, and the acidic pH of the cell medium did not cause the inhibitory effect of the DOX toxicity in HSC-3 cells. At pH 5.6, the higher cell toxicity of DOX@NGO-BBN-AF750 could be explained by the increase of the drug release at the acidic pH and the internalization of DOX@NGO-BBN-AF750 for intracellular utilization of the drugs. Another recent study also reported that there was no significant direct effect in cell viability on liver cancer cells (HepG2) treated under different pH values or treated with DOX under different pH values (58).

### *In vitro* stability of DOX@NGO-BBN-AF750

As shown in **Figure S1** (Supplement Material), we found that NGO began to precipitate after 48 h in 10% FBS, and the precipitation was obvious after 72 h. The precipitation of NGO-BBN-AF750 in 10% FBS could be observed at 72 h. However, DOX@NGO-BBN-AF750 remained highly stable in 10% FBS after 72 h. The increased stability in physiological media may be due to the negative charge on the NGO surface being consumed during the synthesis of NGO-BBN-AF750 and DOX@NGO-BBN-AF750.

## Discussion

OSCC is one of the most common malignant tumors in the oral cavity, accounting for 80%–90% of head and neck cancer (HNCC) (59, 60). The early main treatment is surgical intervention. Chemotherapy/radiotherapy is required when the margin is affected after surgery or metastatic lesions are observed in more advanced cases (61). However, conventional treatments are widely accepted for the treatment and/or management of OSCC. Many oral cancer patients after surgical resection would be disfigured and suffer from long-term effects including fatigue, speech problems, swallowing difficulties, weakness, dizziness, hearing loss, and sinus damage (62). Chemotherapy drugs are prone to systemic side effects due to lack of specificity (63). Innovations in nanomedicine and nanotechnology have led to significant advances in the development of nanomaterials for medical applications such as diagnosis, treatment, and imaging-guided intervention. In the past few years, due to its potential application in oncology, drug delivery systems using nanopolymer or inorganic nanoparticles have been widely studied (14, 64). To achieve an effective cancer treatment system, nanoparticles need to have some characteristics in their surface area and size, and a good biocompatibility. As an important factor, with the increase in surface area, the quantity of anticancer agents that can be attached increases. Furthermore, the size of nanomaterials is also a consideration (14, 65). NGO has a large specific surface area and good biocompatibility and was selected as a drug carrier in this study. Liu et al. explored the graphene capabilities in biological systems for the first time in 2008 (66). Several other groups reported the application of GO in drug delivery and cancer therapy (67, 68).

Conventional chemotherapy faces common problems, such as non-selectivity, rapid clearance, and short half-life of the chemotherapeutic drugs, which would lead to inconsistent bioavailability and require higher drug concentrations (69). Interestingly, the EPR effect of nanoparticles allows the drug carriers to accumulate inside the tumor through passive targeting (70). Further attachment of tumor-targeting vectors on the nanoparticle surface is a potential strategy to enhance the drug delivery through active targeting (70, 71). Kefayat et al. investigated the effect on the radiosensitizing efficacy of the albumin-stabilized gold nanoparticles functionalized with different targeting vectors including folic acid, AS1411 aptamer, glucose, and glutamine on breast cancer models (72–74). The study showed that glutamine- and folic acid-functionalized nanoparticles significantly increased the tumor targeting but did not exhibit any significant advantage over each other. GRPR, a G-protein-coupled receptor, has been proven to be overexpressed on many human tumors (75). A study has been reported on the GRPR-targeted delivery of pDNA or siRNA in GRPR-overexpressing cell lines (76). Mansi et al. recently reviewed the present developments on the GRPR-targeted pharmaceuticals for human tumor imaging (77). Moreover, Honer et al. reported the first ^18^F-labeled BBN antagonist for GRPR-positive tumor positron emission tomography (PET) imaging, which has currently been in a phase I clinical study of patients (78). Wang et al. suggested that GRPR might be a helpful specific target for therapy if combined with folate-bombesin (79). However, few studies had investigated the GRPR targeting potential in human head and neck squamous cell carcinoma. Our previous work (48) demonstrated that NGO-BBN-AF750 was efficiently internalized by HSC-3 cells. In the current study, NGO was first coupled with BBN-AF750 to achieve tumor targeting and visualization and further loaded with anticancer drug DOX. Therefore, DOX@NGO-BBN-AF750 has the potential to be developed into a theranostic drug delivery carrier for diagnosis and treatment of tumor.

Tumor tissues are acidic with the pH value in the range of 6.5–6.8 which is lower than that of normal tissue (pH ~7.4) (80). The pH-sensitive nanocarriers could significantly improve the bioavailability of the delivered anticancer drugs at the tumor site while sparing the normal cells from the cytotoxicity. In this study, the interaction between NGO and DOX is mainly through π–π bond stacking, hydrogen bonding, and hydrophobic interaction, so the pH-sensitive release of DOX can be easily achieved. The results of this work show that the cumulatively released drugs in PBS solution are pH dependent, with 19.8%, 17.7%, and 15.3% after 24 h, at pH 5.6, 6.6, and 7.4, respectively. Furthermore, DOX@NGO-BBN-AF750 showed a dose-dependent and pH-dependent cytotoxicity response on HSC-3 cells. The pH-dependent response on cell viability is related to the pH-sensitive drug release rate of DOX@NGO-BBN-AF750. DOX@NGO-BBN-AF750 showed lower activity than the free DOX in the HSC-3 cell viability experiment, which is attributed to the partial release of DOX from the nanoparticle. Hence, DOX@NGO-BBN-AF750 presents the characteristics ensuring a slow release of DOX from the nanodrug delivery carrier, thereby protecting the drug from degradation, and prolonging the half-life of the drug.

## Conclusion

In this study, we successfully synthesized DOX@NGO-BBN-AF750 nanocomposite by the non-covalent bonding method to couple carboxylated NGO with BBN-AF750 and doxorubicin through π–π and hydrogen bonding. BBN endowed the system for tumor targeting while DOX had therapeutic effects and the nanocarrier presented pH sensitivity. Therefore, the system achieved fluorescence imaging and controlled drug release in cancer cells. This report provides a versatile strategy to achieving targeted and imaging-guided therapy of OSCC. Based on the research, we anticipate that this nanocarrier system may hold a potential for application in OSCC theranostics.

## Data availability statement

The raw data supporting the conclusions of this article will be made available by the authors, without undue reservation.

## Author contributions

Conceptualization, LM, JD, and RL; methodology, FZ and XW; software, RG and YZ; validation, RG and YZ; formal analysis, BL; investigation, RG and YZ; resources, RL, FZ, and LW; data curation, RG and YZ; writing—original draft preparation, RG; writing—review and editing, RG, RL, and LM; visualization, RL, LM, and JD; supervision, LM; project administration, RL; funding acquisition, RL. All authors listed have made a substantial, direct, and intellectual contribution to the work and approved it for publication.

## Funding

This work was partially supported by a Research Project Supported by the Shanxi Scholarship Council of China (2021087), Shanxi Province Basic Research Program (202103021223235), and Fund Program for the Scientific Activities of Selected Returned Overseas Professionals in Shanxi Province (20220020).

## Acknowledgments

We acknowledge the expert input from Prof. Ping Yu, University of Missouri-Columbia, on the interpretation of the FT-IR spectroscopy results.

## Conflict of interest

The authors declare that the research was conducted in the absence of any commercial or financial relationships that could be construed as a potential conflict of interest.

## Publisher’s note

All claims expressed in this article are solely those of the authors and do not necessarily represent those of their affiliated organizations, or those of the publisher, the editors and the reviewers. Any product that may be evaluated in this article, or claim that may be made by its manufacturer, is not guaranteed or endorsed by the publisher.

## Supplementary material

Supplementary Figure 1 The dispersion of nanoparticles after 0, 12, 24, 48 and 72 hours,respectively, in 10% FBS. (A) NGO, (B) NGO-BBN-AF750, and (C) DOX@NGO-BBN-AF750.
