# Supplementary material for: pH-responsive graphene oxide loaded with targeted peptide and anticancer drug for OSCC therapy
Source: Front Oncol. 2022 Aug 3;12:930920. doi: 10.3389/fonc.2022.930920 (PMC9382286; doi:10.3389/fonc.2022.930920)
Supplement: Supplementary Figure 1 — The dispersion of nanoparticles after 0, 12, 24, 48 and 72 hours,respectively, in 10% FBS. (A) NGO, (B) NGO-BBN-AF750, and (C) DOX@NGO-BBN-AF750. [file Image_1.pdf]

Supplementary Material

pH-Responsive Graphene Oxide Loaded with Targeted Peptide and Anti-Cancer Drug for OSCC Therapy

Ran Li[1,2†,*], Ruifang Gao[1,2†], Yingjiao Zhao[1,2], Fang Zhang[2], Xiangyu Wang[1,2], Bing Li[2], Lu Wang[2], Lixin Ma[3,4,*], Jie Du[1,2*]

[1] Department of Preventive Dentistry, Shanxi Medical University School and Hospital of Stomatology, Taiyuan 030001, China

[2] Shanxi Province Key Laboratory of Oral Diseases Prevention and New Materials, Shanxi Medical University School and Hospital of Stomatology, Taiyuan 030001, Shanxi, China.

[3] Research Division/Biomolecular Imaging Center, Harry S. Truman Memorial Veterans' Hospital, Columbia, Missouri 65201, United States

[4] Departments of Radiology, University of Missouri, Columbia, Missouri 65212, United States

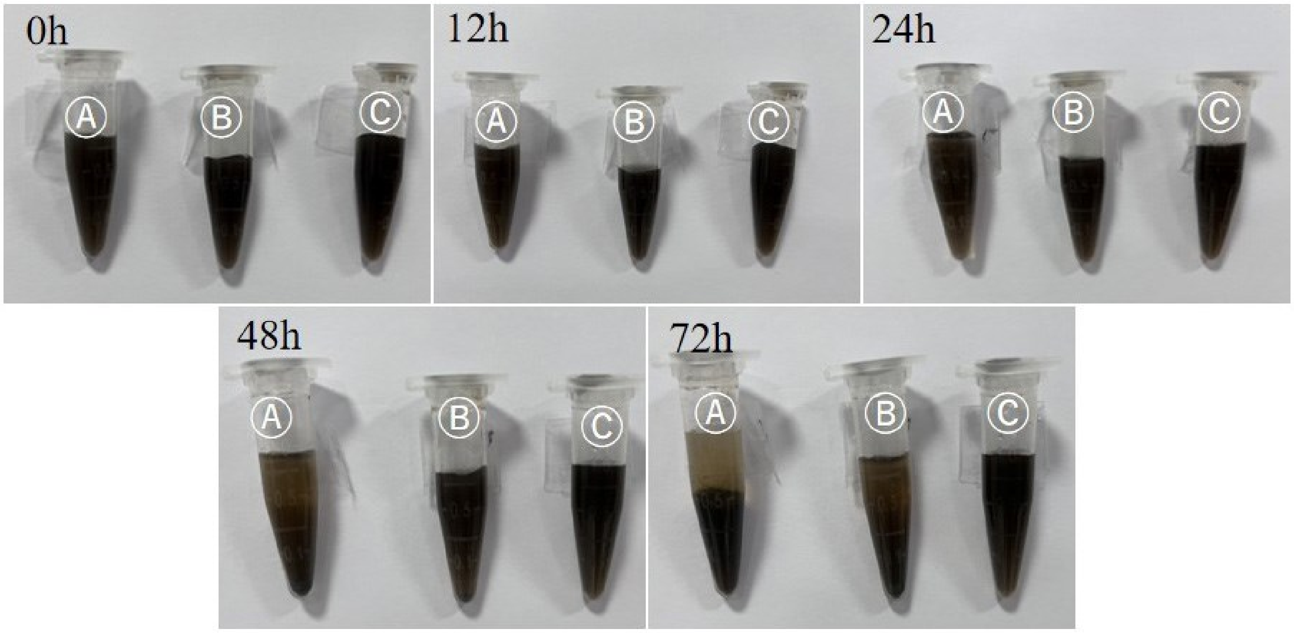

Supplementary Figure S1. The dispersion of nanoparticles after 0, 12, 24, 48 and 72 hours, respectively, in 10% FBS. (A) NGO, (B) NGO-BBN-AF750, and (C) DOX@NGO-BBN-AF750.
